# *In silico* comparative characterization of pharmacogenomic missense variants

**DOI:** 10.1186/1471-2164-15-S4-S4

**Published:** 2014-05-20

**Authors:** Biao Li, Chet Seligman, Janita Thusberg, Jackson L Miller, Jim Auer, Michelle Whirl-Carrillo, Emidio Capriotti, Teri E Klein, Sean D Mooney

**Affiliations:** 1 The Buck Institute for Research on Aging, Novato, CA, USA; 2 Department of Genetics, Stanford University, Stanford, CA, USA; 3 Department of Pathology, University of Alabama at Birmingham, Birmingham, AL, USA

## Abstract

**Background:**

Missense pharmacogenomic (PGx) variants refer to amino acid substitutions that potentially affect the pharmacokinetic (PK) or pharmacodynamic (PD) response to drug therapies. The PGx variants, as compared to disease-associated variants, have not been investigated as deeply. The ability to computationally predict future PGx variants is desirable; however, it is not clear what data sets should be used or what features are beneficial to this end. Hence we carried out a comparative characterization of PGx variants with annotated neutral and disease variants from UniProt, to test the predictive power of sequence conservation and structural information in discriminating these three groups.

**Results:**

126 PGx variants of high quality from PharmGKB were selected and two data sets were created: one set contained 416 variants with structural and sequence information, and, the other set contained 1,265 variants with sequence information only. In terms of sequence conservation, PGx variants are more conserved than neutral variants and much less conserved than disease variants. A weighted random forest was used to strike a more balanced classification for PGx variants. Generally structural features are helpful in discriminating PGx variant from the other two groups, but still classification of PGx from neutral polymorphisms is much less effective than between disease and neutral variants.

**Conclusions:**

We found that PGx variants are much more similar to neutral variants than to disease variants in the feature space consisting of residue conservation, neighboring residue conservation, number of neighbors, and protein solvent accessibility. Such similarity poses great difficulty in the classification of PGx variants and polymorphisms.

## Background

In humans missense variants that introduce amino acid substitutions in proteins have received extensive study as the rich knowledge we have accumulated on protein function and structure greatly facilitates such investigation. One intensive research area in this field currently focuses on identifying and annotating variants associated with important functional or phenotypic consequences [1,2]. In the latter, particularly for disease variant prediction, numerous powerful predictive tools using a variety of feature sets have been proposed. For instance, SIFT [3] largely explored sequence conservation information, PolyPhen2 [4] used physicochemical and crystal structure properties, SNPs&GO included functional information [5], and MutPred [6] integrated a comprehensive list of changes derived from protein sequence. These tools all operate similarly, a classification model based on two curated data sets, e.g., disease and neutral variant data (the latter being usually referred as polymorphism) is constructed, or alternatively, from functional and non-functional variant data. Compared with the many efforts invested in predicting disease variants [7], the prediction of missense PGx variants by similar approaches has not been studied nearly as thoroughly.

PGx variants may affect one's response to a drug through influencing either PK or PD machinery. The clinical procedure for identifying PGx variants usually involves measuring drug concentration and several response parameters within a cohort, such as blood pressure or body temperature, during a period of time [8]. Also PGx variants can be identified from GWAS studies or through candidate gene approaches in large retrospective cohorts. However these approaches are either time-consuming or resource-intensive, and computational alternatives are highly valuable and may aid in characterization of whole genome sequence interpretation [9]. An attempt to build a counterpart to MutPred in making missense PGx variant prediction revealed two issues.

The first issue revolves around the usefulness of the recognized features that have proven to be beneficial in disease-neutral variant classification. One of the salient distinctions between disease and neutral variants is that disease variants generally occur on much conserved sites whereas the positions of neutral variants lack such restriction [3,10]. Therefore, it is not a surprise to see that protein sequence conservation serves as the most important single feature in discriminating disease variants from neutral ones [6]. Additionally, structural information, especially conservation of neighboring residues in 3D protein structures, has been demonstrated to be very useful in classifying catalytic residues [11,12] and other functional sites [13,14]. Unlike disease or other functional variants, PGx variants have undergone much less selective pressure as the corresponding evolutionary environment--modern medicine--is relatively recent. Hence, PGx variants may not possess the same strong sequence conservation patterns as disease variants do.

The second issue is that it is not clear about what variants may constitute a biologically sound non-PGx group. The selection of the negative set is potentially affected by the presence of functional variants among the polymorphisms [15]. The most prominent data repository for PGx variants is PharmGKB (The Pharmacogenomics Knowledgebase; http://www.pharmgkb.org) [16] which has curated about 5,000 variants from more than 900 genes. Only a small portion of these variants is missense. The relatively small coverage of PharmGKB suggests that available PGx variants are less representative and many variants outside PharmGKB at present may exhibit PGx effect in the future studies.

To address the above issues, we collected a set of high-quality and likely causative PGx variants from PharmGKB and performed comparison of PGx with neutral and disease variants.

## Results

### Data description

We applied a rigorous process to search for high-quality and likely causative PGx variants in PharmGKB (see Methods), and identified 55 PD and 71 PK missense variants with high confidence. Due to the small size of these two variant sets, we merged them into one group to increase statistical power in the analysis. One important issue in the classification of functional variants is the selection of an accurate negative set of non-functional ones. At the moment there is no common practice to address this issue. Thus, in this work we consider all the variants annotated in UniProt [17] as "Polymorphism" are functionally neutral. Accordingly, in the rest of the paper we use the word *neutral *to represent the missense variants annotated in UniProt as "Polymorphism".

In our analysis, we used a dataset composed by 126 PGx variants located in 64 proteins, from which we also selected additional 487 polymorphisms and 652 disease variants annotated in UniProt database (version 2013_09). It is noteworthy that 104 (83%) of the PGx variants are labeled as polymorphism in UniProt, suggesting that an annotation of Polymorphism does not necessarily mean not pharmacogenetic. On the other hand, 11 PGx variants from current data are labeled as disease in the same database, suggesting some overlap between disease and PGx variants. We excluded these overlapped variants from neutral and disease variant sets, respectively. The 64 proteins belong to 35 superfamilies and we further grouped them into six classes based on their annotations (Table 1). PGx variants differ from disease variants in the distribution across six protein classes (*r *= 0.40, Pearson correlation coefficient) but show a highly similar trend as neutral variants (*r *= 0.95). Disease variants are most frequently in channel proteins with a density of 51 variants per protein, whereas PGx and neutral variants both are enriched in Cytochrome P450 proteins with much smaller densities (2.7 and 12.5, respectively). Counting number of proteins, we found that 63 proteins have annotated neutral variants, while only 15 are associated with disease variants. Such observation is very similar to what we encountered previously in a much more comprehensive data set [6]. From annotations in UniProt on amino acid modifications, we could only identify five variants out of total 1,265 ones with annotated phosphorylation or methylation sites. As such a sparse coverage presents very limited variance among variants, functional annotations were excluded from further analysis.

**Table 1:** Protein and variant distribution across protein types.

Type	Protein	Variant		
		
		Neutral	Disease	PGx
Enzyme	22	116	130	34
Transporter	14	165	286	34
Cytochrome P450	11	138	47	30
Receptor	10	39	13	17
Channel	3	18	153	4
Others	4	11	23	7
Total	64	487	652	126

### Feature generation from protein structure and sequence

Sequence conservation has been shown to be powerful in discrimination of disease and neutral variants [3,10,18-20]. Here, we measured sequence conservation for each amino acid site by calculating a normalized evolutionary rate from a global multiple species alignment in UCSC genome browser [21]. Because an earlier version of such data had been successfully explored in concentrating on benign with damaging missense variants [10], we followed this approach but used the Rate4Site tool [22] for higher accuracy of conservation estimation. Through searching the Protein Data Bank (PDB) [23], we identified 31 proteins with X-ray structures that covered a total of 416 variants (187 neutral, 174 disease, and 55 PGx). The following features based on sequence conservation and protein structure were derived for 416 variants: average conservation scores of neighboring residues in 3D, number of neighbors, accessibility, and secondary structure. Other features, which could be generated from sequence, include predicted B-value, protein stability, hydrophobic property, and change in molecular weight, where B-value is a normalized B-factor of C-α of each residue and reflects the structural flexibility around it [24]. Two sets of variants were created: a structure data set with 416 variants and 10 features, and a sequence data set with 1,265 variants and six features (see Additional files 1 and 2 respectively).

### Feature difference among three groups of variants

**Figure 1:**
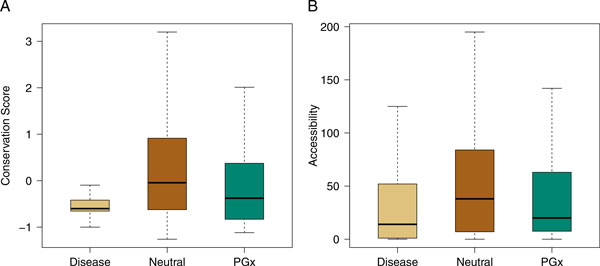
**Comparison of conservation scores and accessibility among disease, neutral, and PGx variants**. In each box-and-whisker plot, the black lines denote medians, boxes with gray background cover from the first to third quantile, and notches connecting to the boxes with dashed lines cover close to 95% interval. **(A)** Disease variants show strongest conservation and fall within a narrow score range; PGx variants are less conservative and the scores disperse in a larger range but less broad than neutral variants do. **(B)** Accessibility is measured by the number of water molecules. Disease variants are the least accessible, and the accessibility distribution of PGx variants is more similar to that of disease variants than to neutral ones, which scatter in a significantly larger range.

Table 2 shows the comparison between the distribution of each feature across three groups of variants, e.g., neutral, disease, and PGx by the Kendall correlation test (a non-parametric statistical method for measuring monotonic relationship between two quantities). Compared to the PGx variants, disease variants occur at the most conserved positions while neutral variants at less conserved ones (Figure 1A). One possible explanation for this observation is that PGx variants were exposed to the environment of drug therapies relatively recently in the evolutionary timeframe and thus have undergone little or very mild selective pressure. Similarly, disease variants have the most conserved neighbors and are located in the most crowded environment, and neutral variants display no conservation in neighborhood in 3D. In contrast, PGx variants stand in the middle on both measures (Figure 1B). Interestingly, disease variants tend to be less solvent exposed than PGx and neutral variants. Such a reverse relationship between the number of neighbors in 3D structure and solvent accessibility may be due to that a residue with more neighbors around it provides less space for solvent molecules to approach. There were no significant associations between variant groups and B-value, protein stability, hydrophobic property, or change in molecular weight. At the sequence level, only conservation displayed significant difference among the three groups of variants and the same gradient trend observed in the structure data set was maintained.

**Table 2:** Features showing different distribution among three groups of variant.

Data set	Feature	Variant			*τ*	*P*-value
				
		Disease	PGx	Neutral		
Structure	Conservation	-0.474	0.000	0.209	-0.22	6.1 × 10^-9^
	ConsNG	-0.316	-0.152	-0.021	-0.21	7.6 × 10^-8^
	NNG	10.0	9.4	8.3	0.18	4.8 × 10^-6^
	ACC	30.4	42.0	52.8	-0.17	1.2 × 10^-5^
Sequence	Conservation	-0.323	0.145	0.340	-0.23	7.4 × 10^-25^

Abbreviations: Conservation, conservation at the variant position; ConsNG, average conservation of 3D neighbors; NNG, number of 3D neighbors; ACC, accessibility in number of water molecules; τ, Kendall correlation coefficient. *P*-value is computed from Kendall correlation coefficient test. The Kendall test has been performed sorting the feature vector according their average values in each class (Disease, PGx and Neutral).

### Three-group discrimination

**Figure 2:**
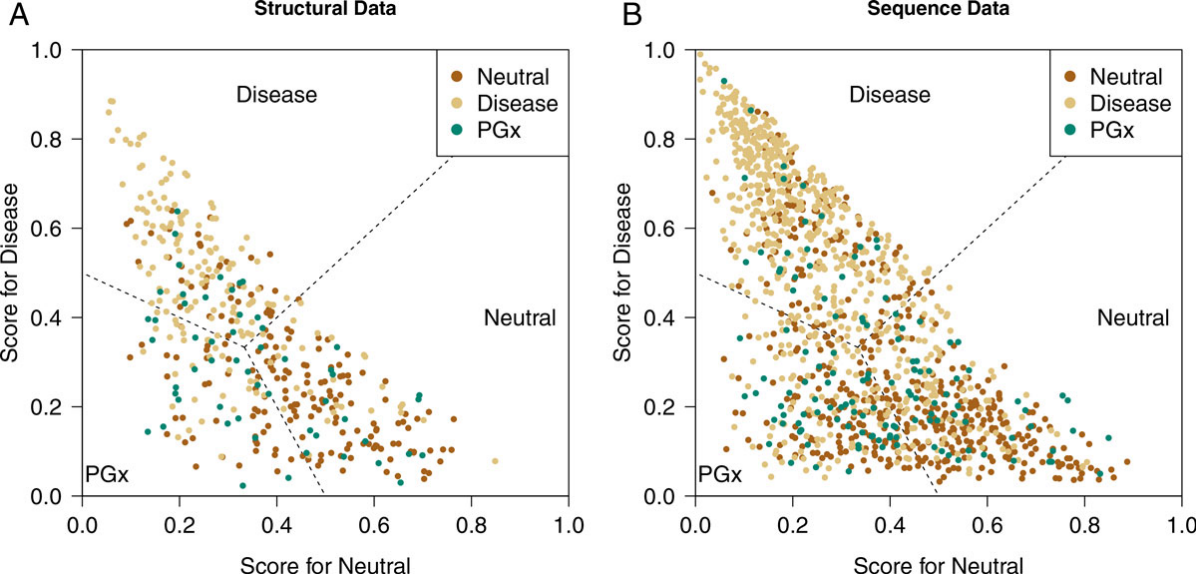
**Spread of three types of variant in the neutral-disease plane**. Dashed lines constitute the prediction decision boundary among three types of variant in the neutral (*x*)-disease (*y*) plane. The intersections in both figures have a coordinate (1/3, 1/3). The decision boundary consists of three lines: *y - x = 0, 1 - 2x - y = 0*, and *1 - x - 2y = 0*. **(A)** Distribution of three groups of variants based on structural data. **(B)** Distribution of three groups of variants based on sequence data.

A random forest was used to evaluate discrimination of neutral, disease, and PGx variants. By assigning larger weights to the minority groups, the classification algorithm was able to achieve more balanced overall accuracy [25]. In the three-group discrimination, we specified group weight approximating the reciprocal of corresponding data size. In the structure data set, an overall error rate of 42%, with 43% in neutral group, 34% in disease group, and 60% in PGx group was observed. Thus, PGx variants are slightly more likely to be misclassified as neutral than as disease (Figure 2A). When we increased the weight on PGx group to increase their penalty on misclassification in random forest, we found a larger increase in the error rate in neutral variants but less in disease variants with the decreasing error rate in PGx variants. This challenge highlights that PGx and neutral variants are mostly overlapped in the given feature space and the differences in conservation, neighbors, and other features are not large enough to provide sufficient classification power for them. In the sequence data, the discrimination was even less successful, although we got triple the data points of the structure data set (Figure 2B).

### Two-group discrimination

As multi-class classification is generally more difficult than two-class classification, we turned to two-group discrimination to see whether a clearer boundary among neutral, disease, and PGx variants could be observed. Table 3 illustrates that by using a similar weighting strategy to that of the three-group discrimination, a more accurate classification in both disease-neutral and PGx-disease tasks was observed, with slightly improved performance from structure data than from sequence data. Once again, the inferior performance observed in PGx-neutral classification signifies more than moderate similarity between them. If we use any of the prediction accuracy measures as an indicator of the similarity between data sets, then we find PGx variants stand between neutral and disease variants.

**Table 3:** Classification performance measurements from two-group comparison (%).

Classification	Sensitivity	Specificity	Accuracy	Precision	*F*-measure	AUC
Structure						
Disease/neutral	76.4	75.4	75.9	74.3	75.4	82.0
PGx/disease	67.3	74.1	70.7	45.1	54.0	78.1
PGx/neutral	58.2	66.3	62.2	33.7	42.7	64.2
Sequence						
Disease/neutral	73.5	68.4	70.9	75.7	74.6	79.3
PGx/disease	68.3	72.4	70.3	32.3	43.9	77.4
PGx/neutral	47.6	63.9	55.7	25.4	33.1	56.9

### Allele frequency of three groups of variants

**Figure 3:**
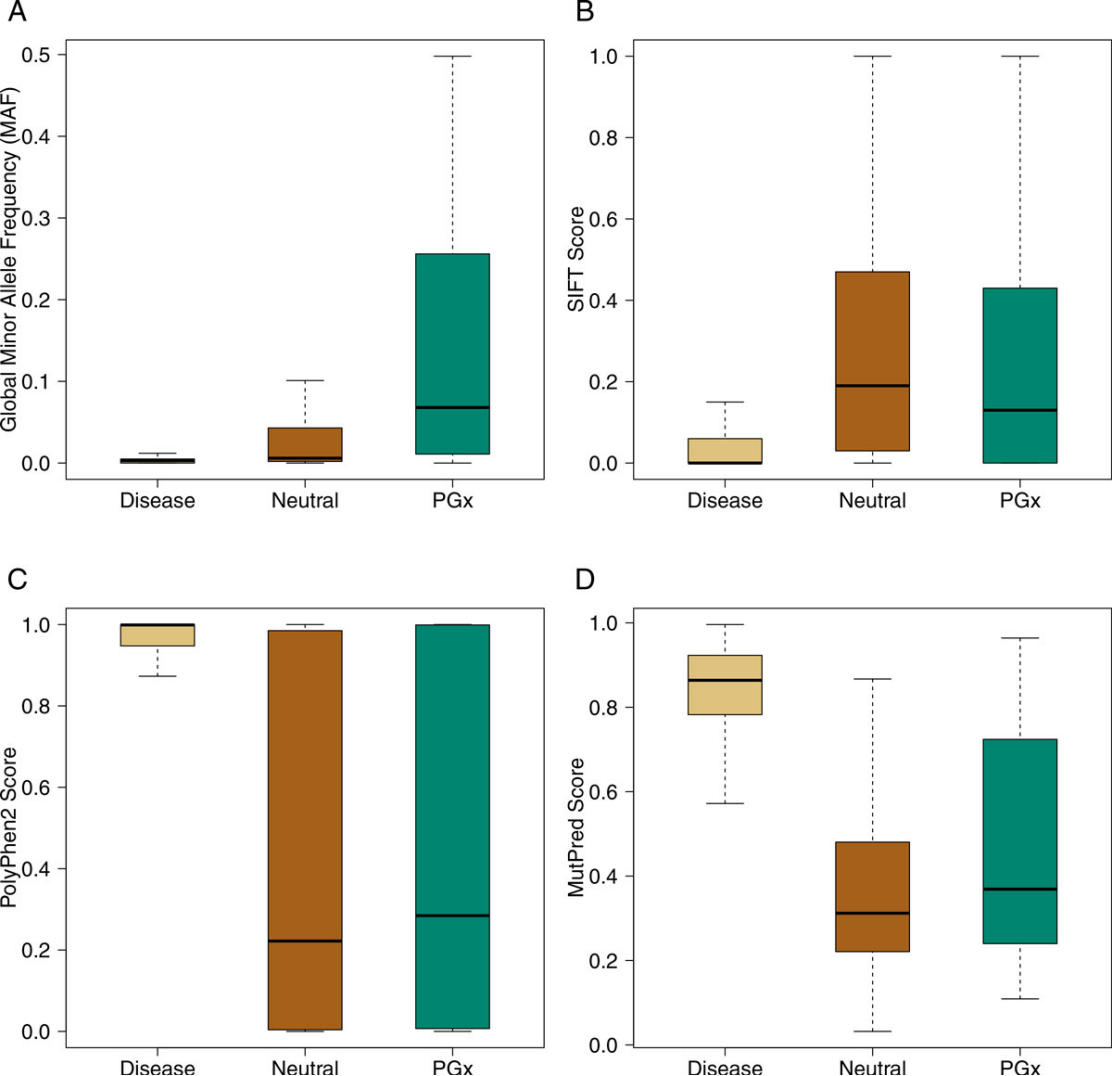
**Comparison of minor allele frequency and pathogenesis among disease, neutral, and PGx variants**. Box-and-whisker representation of minor allele frequency and pathogenesis among three groups of variants. Note that in **(A)** not all variants could find MAF data due to being missing in the 1,092 individuals from the 1000 Genomes Project. **(B)** SIFT uses a threshold of 0.05 to classify variants with scores lower than this value as pathogenetic. **(C)(D)** Both PolyPhen2 and MutPred output probability-like scores measuring pathogenesis of variants.

In addition to interspecies conservation, another way of assessing evolutionary influence on variant sites is through comparing the minor allele frequency (MAF) of the variants across representative human populations. The phase 1 data from the 1000 Genomes Project provided MAF information for 68,300 missense variants identified from 1,092 individuals from 14 populations worldwide [7]. Here we extracted frequency information for all variants from dbSNP annotations provided by the 1000 Genomes Project. Surprisingly, only 4% disease variants could find MAF data, indicating that the most majority of them were not observed in such a global population sample and hence are likely to be rare in the whole population. On the other hand, 57% neutral and 74% PGx variants were observed at least once in the same population sample. Furthermore, the average MAF of disease variants (0.004) is much lower than either neutral (0.061, *P *= 1.6 × 10^-3^) or PGx variants (0.139, *P *= 1.6 × 10^-9^; Figure 3A). Interestingly, PGx variants are significantly more frequent than neutral ones (*P *= 3.5 × 10^-9^) and it remains to verify whether such a trend still holds in larger data sets.

### Comparison of predicted pathogenesis among three groups of variants

Although our sequence and structural feature-based random forest models are less successful in classifying PGx and neutral variants than in classifying disease and the other two groups of variants, it may be informative to bring in some well-established tools for pathogenesis prediction to this task. We then applied three widely-used pathogenesis prediction tools, i.e., SIFT [3], PolyPhen2 [4], and MutPred [6], to all 1,265 variants (see Additional file 3). Unlike PolyPhen2 and MutPred, SIFT outputs for each non-synonymous variant a score measuring the level of tolerance and thus a lower score denotes higher probability of being pathogenetic (Figure 3B). We observed that both neutral and PGx variants are significantly less pathogenetic than disease variants (disease vs. neutral: *P *= 6.3 × 10^-53^; disease vs. PGx: *P *= 6.7 × 10^-13^). Both PolyPhen2 and MutPred generate probability-like score in estimating how likely a variant is to be pathogenetic, and both tools show similar score distributions among disease, neutral, and PGx variants (Figure 3C and Figure 3D), where scores for disease variants are significantly higher than scores for either neutral or PGx variants. Across all three tools, the average score for PGx variants sits between those for neutral and disease variants, and one plausible explanation is that PGx variants could possess certain weak deleterious effects compared to neutral variants but these effects are far less severe than effects of disease variants.

In a similar manner to the random forest models, we tested how accurate each tool is in classification across three settings using its predicted scores and recommended threshold. Although all three tools have seen comparable or better performance in distinguishing disease variants from either PGx or neutral variants, they suffered as the random forest models did in discriminating between PGx and neutral variants (Table 4).

**Table 4:** Classification performance measurements from three tools (%).

Classification	Sensitivity	Specificity	Accuracy	Precision	*F*-Measure	AUC
SIFT						
Disease/neutral	71.1	71.1	71.1	75.1	73.1	77.3
PGx/disease	71.1	60.6	65.8	90.3	79.6	70.5
PGx/neutral						54.8
PolyPhen2						
Disease/neutral	85.3	57.1	71.2	72.7	78.5	76.9
PGx/disease	85.3	54.0	69.6	90.6	87.8	69.9
PGx/neutral						53.8
MutPred						
Disease/neutral	98.5	76.6	87.5	84.9	91.2	95.1
PGx/disease	98.5	61.1	79.8	92.9	95.6	85.9
PGx/neutral						59.1

Classification thresholds between PGx and neutral variants are unknown and thus only AUC was computed for each tool.

## Discussion

From this study PGx variants appear at sites with relatively low sequence conservation and are common in global human populations, and their physicochemical properties resemble those of neutral variants. An appealing explanation for such observations may be that PGx variants have come to be under selective pressure very recently in evolutionary history. The story, however, may be more complex than this as the finial phenotypic effect of PGx variants is not as straightforward as that of disease variants. While typically variants involved in Mendelian diseases cause loss of function and manifest narrow phenotypic spectra, PGx variants have subtler effects on the PK and PD of drugs. For instance, only 6% of disease variants in current study have been annotated with more than one disease, while about half of PGx variants are related to two or more drugs and their effects depend on the very drugs and the conditions they target. Another thread to make things more complex is that the exact effects of a PGx variant may largely depend on the drug dosage and increments in the plasmatic concentration of the drug resulting from such a variant may have divergent effects.

Imbalanced data are common in biomedical research. In our data, the ratio of the number of variants from the most frequent group to the least frequent is 3.4 in the structure data set and 5.2 in the sequence data set. Without tuning the classification algorithms, which by default assume a balanced training set, we observe a higher error rate in the minority group. Unbalanced performance on the two subsets is due to the higher contribution of the most abundant class to the overall accuracy. In two-group discrimination, we achieved approximately balanced sensitivity and specificity across six classification settings by properly assigning higher weight to the PGx variant group. The benefit of such weighting was more noticeable in three-group discrimination. Without modification to the default random forest, the PGx group in the sequence data is almost completely misclassified, rendering the entire classification approach far less sensible.

In current study sequence conservation and structural information have shown limited capability in discriminating PGx variants from neutral variants. One approach to improve classification without a dramatic increase in the size of the PGx variant dataset might lie in integrating novel features, such as those derived from 3D structure. One of such potential alternative features is free energy change (∆∆G) caused by a missense variant, which can be well estimated by FoldX [26] through protein structural calculations. A recent study in the context of dynamic pathway analysis found that ∆∆G calculated from FoldX is directly linked to cell growth and such link can be expressed in ordinary differential equations accurately [27]. Thus ∆∆G may serve as an indicator to the potential influence a variant could cause. Comparing with the classification of disease and neutral variants, the similar problem between PGx and neutral variants is less amenable to otherwise well-established methodologies. While structure-based features are not as accessible as those derived from sequences, we did observe that they help improve the performance of classification slightly. This observation may in turn suggest that exploring more structure-related feature might prove to be profitable.

## Conclusions

We have determined that PGx variants possess a higher degree of similarity to neutral genetic polymorphisms than to disease variants in the feature space consisting of residue conservation, neighboring conservation, number of neighbors, and accessibility. Such similarity poses a great difficulty in the classification of PGx and neutral variants. Future studies focusing on predicting PGx variants may need a better definition of non-PGx variants or to explore novel features outside those that have been demonstrated to be useful in disease-neutral variant classification.

## Methods

### Data collection

All protein variants annotated as associated with a PGx trait were extracted from PharmGKB (http://www.pharmgkb.org). Two investigators then examined each variant further through curating the original publication from PharmGKB for a causative relationship between the variant and PK/PD effects. The selected variants were then additionally confirmed by PharmGKB scientific curators.

### Calculating residue conservation

We downloaded from UCSC genome browser multiple alignments of 45 vertebrate genomes with Human that includes amino acid alignment from hg19/GRCh37 RefSeq genes (http://hgdownload.soe.ucsc.edu/goldenPath/hg19/multiz46way/alignments/refGene.exonAA.fa.gz) [21]. We then applied the tool Rate4Site [22] to the multiple protein sequence alignment to estimate the relative evolutionary rate for each site, with the supplied phylogenetic tree that was used to calculate PhastCons scores in UCSC genome browser at the nucleotide level. The mapping between RefSeq and UniProt sequences was carried out by the global alignment program ggsearch in FASTA [28].

### Feature generation

Three-dimensional structures were downloaded from the PDB [23]. For each protein, we chose the structure with the largest coverage or the highest resolution if multiple structures with the same coverage were available, and then searched for neighboring residues and extracted secondary structure for each variant based on such structure. To define the neighboring residues to a given amino acid in a protein structure we followed the definition of Cilia & Passerini [11]. There every residue is first represented by the centroid of its side-chain heavy atoms and then any residue falling in a sphere with a radius of eight angstroms centered on the given residue is defined as a neighbor. For glycine, the alpha-carbon was used as the centroid. Secondary structure and accessibility (in terms of number of water molecules) of each variant were directly read from the output of DSSP [29,30] as applied to the corresponding PDB file. The PDB residue sequences were mapped to the UniProt sequences as we did in mapping the RefSeq and UniProt sequences. Protein stability changes due to variants were estimated by MUpro [31] which estimates both energy changes and the reliability of underlying prediction.

### Statistical analysis

All computation was carried out in R (http://www.r-project.org). The Kendall correlation coefficient and test was computed by the function cor.test, and Wilcoxon rank sum test was computed by the function wilcox.test. Area under the curve (AUC) was calculated through the R package ROCR [32]. Classification performance measurements are defined below:

Sensitivity = TP/(TP + FN)

Specificity = TN/(TN + FP)

Accuracy = (Sensitivity + Specificity)/2

Precision = TP/(TP + FP)

*F*-measure = 2 × Precision × Sensitivity/(Precision + Sensitivity)

where abbreviations represent

**Table 1:** 

Class	Predicted Positive	Predicted Negative
True Positive	TP	FN
True Negative	FP	TN

### Weighted random forest

The source code for implementing the random forest was obtained from Leo Breiman's web site (http://www.stat.berkeley.edu/~breiman/RandomForests/cc_examples/prog.f) and compiled as a single program for each experiment by the GNU Fortran compiler with the following parameters: -std=legacy -ffixed-form -ffixed-line-length-none -fno-range-check. The out-of-bag predictions from each experimented were weighted internally according to supplied control parameters and normalized to produce prediction scores, which were then used in computing individual measurements.

## Competing interests

The authors declare they have no conflict of interests in relation to this SNP-SIG issue article.

## Authors' contributions

SDM conceived the study. CS, JT, JLM, EC, JA, MWC, and TEK collected data. BL, CS, and SDM analyzed the data. BL and SDM drafted the manuscript with edits provided by EC, MWC, and TEK.

## Supplementary Material

Additional file 1 Features for the structure data set This is the data file corresponding to the structure data set used in the paper. It is in the csv format with 416 variants and 14 columns.

Additional file 2 Features for the sequence data set This is the data file corresponding to the sequence data set used in the paper. It is in the csv format with 1,265 variants and eight columns.

Additional file 3 MAF and pathogenesis predictions This file contains minor allele frequency extracted from dbSNP and pathogenesis predictions from SIFT, PolyPhen2, and MutPred. Not all variants have MAF available. SIFT predictions for some variants are missing due to the conflict in variant definition between dbSNP and UniProt.

## Declarations

The publication costs for this article were funded by a grant from NIH R01 LM009722 (PI: Mooney).

This article has been published as part of *BMC Genomics *Volume 15 Supplement 4, 2014: SNP-SIG 2013: Identification and annotation of genetic variants in the context of structure, function, and disease. The full contents of the supplement are available online at http://www.biomedcentral.com/bmcgenomics/supplements/15/S4
